# Correction: Mass spectrometric detection of fleeting neutral intermediates generated in electrochemical reactions

**DOI:** 10.1039/d1sc90175c

**Published:** 2021-08-16

**Authors:** Jilin Liu, Kai Yu, Hong Zhang, Jing He, Jie Jiang, Hai Luo

**Affiliations:** School of Environment, School of Marine Science and Technology (Weihai), Harbin Institute of Technology Weihai Shandong 150090 China jiejiang@hitwh.edu.cn; State Key Laboratory of Urban Water Resource and Environment, Harbin Institute of Technology Harbin Heilongjiang 150090 China; School of Chemistry and Chemical Engineering, Harbin Institute of Technology Harbin Heilongjiang 150001 China; Beijing National Laboratory for Molecular Sciences, College of Chemistry and Molecular Engineering, Peking University Beijing 100871 China hluo@pku.edu.cn

## Abstract

Correction for ‘Mass spectrometric detection of fleeting neutral intermediates generated in electrochemical reactions’ by Jilin Liu *et al.*, *Chem. Sci.*, 2021, **12**, 9494–9499, DOI: 10.1039/D1SC01385H.

The authors regret that the details for ref. 15 and 17 were inadvertently swapped in the original article. The correct versions of ref. 15 and 17 are given below as ref. 1 and 2, respectively.

The Royal Society of Chemistry apologises for these errors and any consequent inconvenience to authors and readers.

## Supplementary Material

## References

[cit1] Zhang H., Qiao L., Wang W., He J., Yu K., Yang M., You H., Jiang J. (2020). Anal. Chim. Acta.

[cit2] Yu K., Zhang H., He J., Zare R. N., Wang Y., Li L., Li N., Zhang D., Jiang J. (2018). Anal. Chem..

